# Animal Welfare Implications of Digital Tools for Monitoring and Management of Cattle and Sheep on Pasture

**DOI:** 10.3390/ani11030829

**Published:** 2021-03-15

**Authors:** Anders Herlin, Emma Brunberg, Jan Hultgren, Niclas Högberg, Anna Rydberg, Anna Skarin

**Affiliations:** 1 Department of Biosystems and Technology, Swedish University of Agricultural Sciences, P.O. Box 190, 23422 Lomma, Sweden; 2 Djurskyddet Sverige, Hammarby Fabriksväg 25, 12030 Stockholm, Sweden; emma.brunberg@djurskyddet.se; 3 Department of Animal Environment and Health, Swedish University of Agricultural Sciences, P.O. Box 234, 53223 Skara, Sweden; jan.hultgren@slu.se; 4 Parasitology Unit, Department of Biomedical Sciences and Veterinary Public Health, Swedish University of Agricultural Sciences, P.O. Box 7036, 75007 Uppsala, Sweden; niclas.hogberg@slu.se; 5 Division Bioeconomy and Heath, Agrifood and Biosciences, RISE Research Institutes of Sweden, P.O. Box 7033, 75007 Uppsala, Sweden; anna.rydberg@ri.se; 6 Department of Animal Nutrition and Management, Swedish University of Agricultural Sciences, P.O. Box 7024, 75007 Uppsala, Sweden; anna.skarin@slu.se

**Keywords:** animal welfare, cattle, monitoring, precision livestock farming, sensor, sheep, virtual fence

## Abstract

**Simple Summary:**

Monitoring the welfare of cattle and sheep in large pastures can be time-consuming, especially if the animals are scattered over large areas in semi-natural pastures. There are several technologies for monitoring animals with wearable or remote equipment for recording physiological or behavioural parameters and trigger alarms when the acquired information deviates from the normal. Automatic equipment allows continuous monitoring and may give more information than manual monitoring. Ear tags with electronic identification can detect visits to specific points. Collars with positioning (GPS) units can assess the animals’ movements and habitat selection and, to some extent, their health and welfare. Digitally determined virtual fences, instead of the traditional physical ones, have the potential to keep livestock within a predefined area using audio signals in combination with weak electric shocks, although some individuals may have difficulties in responding as intended, potentially resulting in reduced animal welfare. Remote technology such as drones equipped with cameras can be used to count animals, determine their position and study their behaviour. Drones can also herd and move animals. However, the knowledge of the potential effects on animal welfare of digital technology for monitoring and managing grazing livestock is limited, especially regarding drones and virtual fences.

**Abstract:**

The opportunities for natural animal behaviours in pastures imply animal welfare benefits. Nevertheless, monitoring the animals can be challenging. The use of sensors, cameras, positioning equipment and unmanned aerial vehicles in large pastures has the potential to improve animal welfare surveillance. Directly or indirectly, sensors measure environmental factors together with the behaviour and physiological state of the animal, and deviations can trigger alarms for, e.g., disease, heat stress and imminent calving. Electronic positioning includes Radio Frequency Identification (RFID) for the recording of animals at fixed points. Positioning units (GPS) mounted on collars can determine animal movements over large areas, determine their habitat and, somewhat, health and welfare. In combination with other sensors, such units can give information that helps to evaluate the welfare of free-ranging animals. Drones equipped with cameras can also locate and count the animals, as well as herd them. Digitally defined virtual fences can keep animals within a predefined area without the use of physical barriers, relying on acoustic signals and weak electric shocks. Due to individual variations in learning ability, some individuals may be exposed to numerous electric shocks, which might compromise their welfare. More research and development are required, especially regarding the use of drones and virtual fences.

## 1. Introduction

In modern livestock production systems, animals are largely kept indoors or in small enclosures. Pasture-based production systems are decreasing as the demands for high profitability increase. However, pasture-based systems usually offer better hygiene than indoors, provide animals with a softer surface than the commonly used concrete in buildings and allows them to perform natural behaviours, without serious restrictions on their movements. Such conditions have many positive animal welfare effects that meet common consumer expectations [1]. In addition, grazing can be beneficial for biodiversity [2], soil preservation [3] and carbon sequestration [4]. It is good farming practice to regularly inspect the animals’ health and welfare in a pasture, which is also regulated by law in some countries. In Sweden, for example, animals should be inspected at least once daily.

The automated inspection or monitoring from a distance by the use of digital technologies might reduce the labour substantially. In fact, it has been suggested that such technologies would allow farmers to monitor and manage animals more intensively than otherwise, resulting in a higher efficiency in production, lower environmental impact and improved animal welfare [5]. Technological developments in the last two decades have led to several digital applications in the livestock sector. Since environmental conditions outdoors vary in terms of weather, climate, physical conditions, vegetation and geography, the transfer of data over long distances can be technically challenging. Most of the existing sensor technology in agriculture was developed for indoor use. New developments of remotely controlled unmanned aerial vehicles (UAV), commonly called drones, have further increased the possibilities for farm animal monitoring and management.

Sensors record more or less continuously around the clock, and the information acquired can be much more detailed than a single manual inspection per day. The use of sensors thus might have the potential to promote grazing in large pasture areas with an increased monitoring of factors indicating the animals’ welfare status. Nevertheless, sensors can generate objective information about events in the environment or the animals’ conditions (activity, physiology, etc.), but human judgement is often needed to interpret the data, and relevant actions are required to secure the animals’ welfare. An essential question is therefore whether, and to what extent, digital technology can replace manual inspections. A combination of automated digital monitoring and manual follow-up inspections may prove to be a reasonable compromise. Moreover, some applications of wearables and digital technologies may also influence the individual animals in a way that can compromise their welfare.

This systematic review addresses the use of digital technologies for health and welfare monitoring and the management of livestock kept in large pasture areas. The review is mainly focused on cattle (*Bos taurus*) and domestic sheep (*Ovis aries*), but most of the information is applicable to other livestock species as well. This review includes the use of digital technology and new tools for the management of animals, including animal welfare monitoring with the help of drones and virtual fences, as these include sensors and automated responses.

A systematic literature search in English and German was performed in Web of Science Core Collection, CAB Abstracts^®^ and Scopus from all available publication years. The following search string was used: (cattle* OR bovin* OR beef* OR *cow* OR *calf* OR *calves OR *sheep* OR ewe* OR ovine OR lamb* OR *rind* OR *kuh* OR *kalb* OR *kälber* OR *schaf* OR *lamm*) AND (outdoor* OR rangeland* OR enclosure* OR pasture* OR graz* freerange* OR free-range OR “free range” OR *draussen* OR gehege* OR weide* OR freiland*) AND (“new techni” OR “inventive techni” OR infrared* OR drone* OR *positioning* OR gps* OR “invisible fenc” OR “wireless fenc” OR “virtual fenc” OR *accelero* OR *logger* OR *thermograph* OR *telemetr* OR sensor OR sensors OR “neue techni” OR “erfinderische techni” OR *infrarot* OR *drohne* OR “virtueller zaun”) AND (“precision livestock” OR *vigilanc* OR *monitor* OR *surveill* OR control* OR contain* OR *präzisionsviehhaltung* OR *wachsamkeit* OR *überwachung* OR *positionierung* OR “unsichtbarer zaun” OR “unsichtbare zäune” OR *kontroll* OR *eindämm*). The search resulted in 532 references after duplicate removal. From these, relevant references were chosen to be used for the review. They were supplemented with references already known to the authors, resulting in 148 references in total.

## 2. Use of Digital Technologies in Livestock Production

### 2.1. Precision Livestock Farming

The use of sensor technologies in livestock production, often called Precision Livestock Farming (PLF), is a relatively new phenomenon starting with the development of the milking robot in the 1990s. PLF involves modern information technology (IT) in order to provide farmers with information for the control of the production process. PLF is generally defined as a management system that offers continuous, automatic monitoring and control of animal behaviour, health, welfare, production and reproduction, as well as environmental impact of the production, in real time [6,7]. By continuous monitoring of the animals, the farmer can immediately detect changes that may indicate that something is wrong [6,8]. PLF has the potential to completely or partially replace direct inspections on-site once or several times a day.

**Figure 1:**
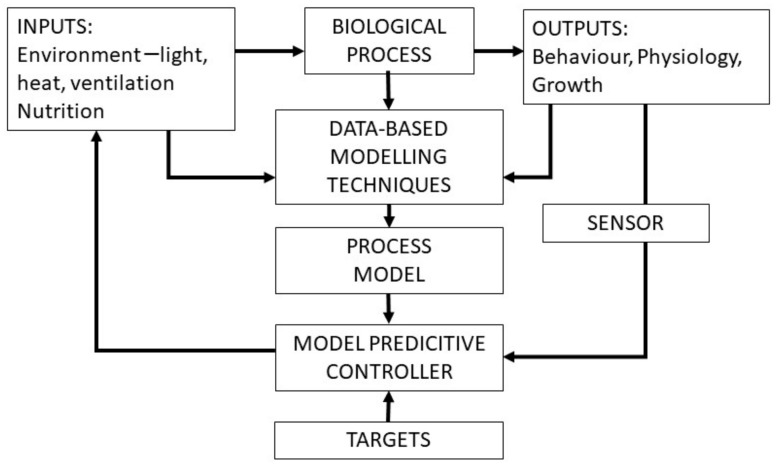
Model of Precision Livestock Farming showing the principal flow of data, the process control where real-time results are compared with a target value, followed by a control function that determines if the information from the sensor matches the target value [6].

In recent years, technological advances have made it feasible to receive accurate data from affordable sensors and instruments, including cameras, microphones, accelerometers (sometimes combined with a gyroscope), thermometers, conductivity meters and devices to determine the physical location. The data are transmitted to computers, where they can be processed and interpreted into useful information, such as identifying animals not following an expected pattern (Figure 1). The Internet of Things (IoT) solutions and several other technologies can be used for wireless communication, data processing and storage. The information obtained can be used to guide some form of automatic or manual action.

### 2.2. Accuracy and Reliability of PLF Systems

The accuracy of a PLF system, i.e., if the abnormalities in animal behaviour, physiology and health are detected correctly, is assessed by several methods depending on the technology involved. The positive predictive value (PPV) is the probability that a positive system output or alarm corresponds to a true state or event. Conversely, the sensitivity (Se) is the probability that a true state or event is followed by a positive system output or alarm, while the specificity (Sp) is the probability that the absence of a true state or event does not produce a positive system output or alarm. Measures to increase the sensitivity of the system by lowering the alarm threshold necessarily can lead to more false positive outputs due to the lowered specificity. The relationship between sensitivity and specificity for different threshold values can be visualised with a Receiver Operating Characteristic (ROC) curve, and the ability of the system to combine a high sensitivity with a high specificity can be calculated as the area under the ROC curve [9]. Other common statistical quality measures are the Pearson or Spearman correlation coefficients (*r* and *ρ*, respectively), the concordance correlation coefficient (CCC), which expresses the degree of linear relationship between two continuous quantities (actual state and sensor signal), and the coefficient of determination (R^2^), which is the proportion of variation of a studied quantity (the actual state) that can be explained by one or more other variables (the sensor signals). The resolution (i.e., the degree of perceptible detail) of a camera system can be expressed as the size of the minimum distinguishable object in the terrain. Furthermore, in an image analysis, the term “hit rate” is used to describe the ability to accurately recognise objects in images.

So far, there is no general agreement or accepted methodology to statistically validate the different sensors or PLF systems, nor to determine what is required for a sensor or system to be considered validated, which makes comparisons of different sensor systems difficult. The introduction of standardised validation protocols would therefore be helpful [10]. Guidelines for evaluating sensor technologies are currently being prepared by the International Committee for Animal Recording [11].

Reliability is not only about correct measurements. There is a risk that advanced technology for animal supervision could completely cease to function, which would also compromise the reliability. We have not found any systematic information on the practical operational reliability of the various commercially available systems for monitoring farm animals, nor have we found information about the presence of alarms for technical faults, although such events occur occasionally.

## 3. Sensor Technologies

A sensor detects conditions or events in the environment or the animal, for example, by measuring the physical, physiological or behavioural indicators or changes in animal management (feeding, bedding and milking), and can provide information about the animal’s condition, e.g., its health, to help the caretaker assess the need for corrective action [12,13].

Sensors can be animal-based or non-animal-based. Animal-based sensors are attached to the animal on ear tags, collars, leg straps or internal boluses or implants. In contrast, non-animal-based sensors are located in the animal’s vicinity—for example, in cameras or automatic scales. In dairy production, there is a variety of commercially available sensors that can be used for animal health and welfare monitoring. They are usually developed for use in indoor systems, but the development of new sensor technologies and infrastructure for data transfer enables their gradual application in grazing animals (Table 1).

### 3.1. Sensors to Measure Physiology

Body temperature can be monitored in order to identify health disorders, changes associated with reproduction, heat stress and hypothermia. There are a number of commercial body temperature sensors, where the most common ones are attached to an ear tag or given in the form of a bolus. Changes in the ambient temperature and the incorrect attachment of ear tags tend to reduce their reliability [15].

Feed and water intake, as well as changes in the rumen microflora, can be detected by rumen boluses. Temperature changes that occur when the animal drinks can be used to calculate their daily water intake and detect if the animal has not been drinking for a certain period of time [16]. Commercially available rumen boluses (a sensor built into a container administered to the rumen) with pH sensors can be used to monitor the condition of the rumen and health disorders leading to a decrease in pH [17], often associated with inadequate feeding routines. In addition, changes in pasture access and composition can be detected if they affect the reticuloruminal pH [18]. Several studies have detected subacute rumen acidosis using commercially available boluses that measure the pH and temperature [19,20].

Implants have been shown to accurately record heart rates in free-grazing sheep [21]. However, for a wider agricultural use, real-time data transfer needs to be developed for this method to become commercially applicable.

### 3.2. Sensors to Measure Behaviour

Changes in behaviour, such as activity, feed intake or rumination, can be linked to normal conditions such as heat and preparations for calving but, also, to disease and pain. A change in animal behaviour may not always be linked to a particular condition but may indicate the need for group management. The continuous monitoring of behaviours makes it possible to identify not only diseased animals but, also, animals at risk of developing health disorders [22]. Commercially available accelerometers, mainly 3D, are usually attached to neck collars, leg straps or ear tags but can also be incorporated into boluses. Depending on where the sensor is located, different behaviours can be related to the accelerometer signals, and thus, the behaviours be quantified. Sensors attached around the neck or in the ear of the animal often provide information on the activity alone, i.e., the total amount of movement [23]. Sensors attached around the animal’s legs can also provide information on the number of steps, lying and standing times and the number of lying bouts. There are a number of techniques for measuring rumination and feeding behaviours, of which the most common are 3D accelerometers, pressure gauges and microphones [24,25,26,27,28]. Commercially available systems mainly use 3D accelerometers and microphones. Depending on the system, they can provide information on the rumination time and number of chewing movements, as well as feed and water intakes [29]. Duncan and Meyer [30] showed that 3D accelerometers can be used to indicate that calving has begun.

Sensor systems can detect a variety of infectious diseases, metabolic disorders and lameness. For example, sensors have been shown to detect changes in the locomotion and lying and/or feeding behaviours in cattle or sheep affected by mastitis [31,32,33], metritis [34], ketosis [35], lameness [36,37,38,39], respiratory disease [40], gastrointestinal parasites [41,42,43,44,45] or a clinical disease in general [46]. Several of the conditions can also be detected at an earlier stage than by manual monitoring, even before the onset of clinical signs.

The major obstacles to a more general use of sensor-aided monitoring in large and remote pastures are the limitations in data transmission and energy supplies. A few systems marketed for use in large pastures have been validated scientifically (Table 2). It is worth noting that these systems were primarily designed for use in housed dairy herds.

## 4. Camera Technologies

Fixed cameras providing still images or videos can be used for small-scale animal inspections. Most applications with fixed cameras are mounted indoors [53]. Cameras mounted on remotely controlled drones may be used for large-scale surveillance. Drones additionally provide overviews of the landscape, which may prove useful in, e.g., habitat selection studies. If the goal is surveillance of an entire herd, cameras can be a cost-effective alternative to collars with positioning receivers, which are expensive and need to be mounted on a large number of animals in order for the surveillance to provide useful information. However, extracting useful information from drone images can be a time-consuming and costly manual task [54]. The future development of vision systems for automatic image processing to automatically and accurately detect and track the individuals of the species in question is crucial for the drone monitoring of animal behaviours and health. From indoor studies, it has been shown that social interactions can be detected by the use of top-mounted cameras [55], which suggests that this could also be done from drone cameras.

Camera systems suitable for animal welfare monitoring are also used in combination with other technologies. Ren et al. [56] combined a multicamera video recording system to detect standing and lying behaviours of sheep with an ultrawideband real-time location system (UWB RTLS). A sensor fusion system was created by combining the animal identity and location (UWB RTLS) with a behaviour monitoring system. The system was based on an earlier study [57] where a similar real-time system was presented for tracking dairy cow behaviours in a semi-open free-stall barn.

In a comparative study of position accuracy of images from drones and collars with positioning receivers, the drone system was superior, with a position accuracy of 1–3 m vs. 26 m [58]. The studied rhinoceros did not appear to be affected by the presence of drones, which accords with the observations of caribou in captivity [59]. Other studies have shown that wildlife may be stressed by drones [60], while some report that they gradually habituate to them [61,62]. Schroeder et al. [60] found that small groups of guanacos were the least affected while the monitoring ability was maintained if drones flew at low speeds (2–4 m/s) 180–200 m over them. The effect of drones on domesticated animals’ behaviours and welfare needs further investigation.

Drones equipped with cameras are increasingly used to count animals and study animal behaviours in the landscape [59]. Algorithms for interpreting the acquired data are constantly improving and have the potential to outperform the human eye [63]. In a wildlife sanctuary in Namibia, with over 3000 animals of 20 different species, drones were used to identify large mammals in their natural environment [64]. This technology is especially useful when counting large numbers of animals over large areas, although false alarms, when objects in the landscape are identified as animals, may be a problem.

Depending on the species studied, different behaviours can be observed. In a study of caribou in Canada, cameras on drones were used to picture and film the animal movement patterns and interactions [65]. Age and, to some extent, gender proved to be decisive for the interactions of an individual with the rest of the herd. Adult animals followed one another, while the calves sought more direct contact with the adults instead of just following in their tracks [65]. Similar methods were also applied in a study of wild horses during the formation of harem groups [66].

The amount of data that can be collected with drones depends, in part, on the area it is possible to cover per flight (as determined by the range and other characteristics of the drone) and, partly, on the system’s ability to detect the object to be studied (the camera’s image resolution). Therefore, the selected altitude is often a trade-off between a high-enough image resolution and the size of the covered area.

A camera or sensor with a low resolution, limited sunlight, dense vegetation and lack of contrast between the surroundings and the study objects, for example, due to shadows, can present difficulties. In most studies conducted with drones and animals in pastures, single-colour images are analysed. This is usually sufficient when locating cattle. Mulero-Pázmány et al. [58] found that both adult cattle and calves could be distinguished from other ungulates, such as wild boars and deer, by using a commercial 11-Mbit pixel camera at an altitude of 100 m. Depending on the density of the vegetation, the number of animals could be slightly over- or underestimated.

Thermal cameras measure the amount of infrared radiation (heat) emitted from an animal’s body surface and may distinguish animals in the dark, making it possible to monitor animals at night [59]. Light conditions, shade or dense vegetation are minor problems for drones equipped with thermal cameras. However, such systems may have difficulty in locating animals if the temperature difference between the animal and the surrounding environment is not large enough [67]. The amount of reflected heat radiation from surfaces largely depends on whether they are exposed to direct or indirect sunlight, e.g., in the middle of the day in direct sunlight, vegetation may reflect thermal radiation similar to that of a living animal [67]. The radiation emitted by animals captured by a thermal camera is not directly related to the animal’s actual body temperature, as the reflected heat depends, among other things, on the insulating effects of skin, fur and more.

Novel analytical methods that combine already published algorithms for machine learning with thermal camera images acquired from drones have been able to automatically detect koalas outdoors more accurately than manually analysed images in terms of both the root mean square error and mean absolute error in a comparable amount of time [68]. The time required for the automatic method mainly consisted of the computer processing time. The studied koalas could be correctly recognised in the drone images despite significantly more complicated terrain, a larger covered area and a lower concentration of animals than in previously published studies. The koalas could also be distinguished from other heat-emitting objects such as humans and kangaroos.

There is a rapid development of image analysis algorithms for locating and identifying animals in an outdoor environment. However, locating animals is a much more common practice than identifying them. Andrew et al. [69] used video sequences acquired from drones to create algorithms that could find and individually identify Holstein-Friesian cows, exploiting their coat pattern uniqueness, in a pasture. In a herd of 23 cows, the animals could be found and identified up to 98% (Se) in over 46,000 frames originating from 34 video clips. This is reliable enough to assist the existing tagging methods in situations with few animals and limited vegetation.

Images of grazing cattle and sheep captured by cameras mounted on quadcopters [70] were used, together with a deep-learning technique Mask Region Based Convolutional Neural Networks (R-CNN) [71], for livestock recognition and counting. The authors examined the effects of different densities and various numbers of training sessions on the classification and counting of species to optimise the model. The proposed system could classify the livestock as cattle or sheep with a sensitivity of 96% and estimate the number of cattle and sheep to within 92% of the visual ground truth and provide the potential of biometrics and welfare monitoring in the animals in real time. The model was tested previously on images of beef cattle by the Danish [72]. Xu et al. [73] evaluated the algorithm and achieved a good performance for the detection of animals.

Drones equipped with cameras show great potential for the supervision of animals kept in large pastures, although the technology must be further developed for more demanding conditions, e.g., night darkness and dense vegetation. The technology to automatically monitor and analyse behaviours and health is being developed rapidly and is crucial for economically feasible animal welfare monitoring in pastures. Just counting animals is not enough, but the system must be able to identify animal postures and distinguish healthy postures from unhealthy ones. Automatic monitoring includes autonomously operating drones without human supervision. However, to our knowledge, there is no research on animal surveillance using drones flying out of sight.

## 5. Positioning Technologies

### 5.1. RFID

Animals can be positioned wirelessly using radio communication. Radio Frequency Identification (RFID) was originally developed for identification purposes, but there are now several different types of positioning and tracking systems based on RFID, and it has become the most commonly used wireless positioning technology. A simple low-frequency RFID system consists of a reader and a transponder (tag), as well as software that converts data on the tag into useful information. RFID transponders can be hidden in ear tags, collars, injected in the neck or orally administered boluses. Schwartzkopf-Genswein, Huisma [74] validated GrowSafe, a commercial RF system for monitoring feeding patterns of feedlot cattle, using three methods: (1) comparing GrowSafe and video methods, (2) comparing bunk attendance data provided by two separate RF transponders carried by a single animal and (3) documenting the relationship between bunk attendance and actual feeding time. GrowSafe was considered to be a valuable tool for documenting the bunk attendance patterns of feedlot cattle. However, factors related to the construction of feedlot pens, like nongrounded (looped) metal panels, may introduce errors into the registered animal presence within the area around the feed bunk. Voulodimos, Patrikakis [75] described a platform for livestock management based on RFID-enabled mobile devices. The platform uses rewritable tags for information storage, where basic information about the animal (e.g., behaviours directed towards other animals) can be stored without the need for contacting the related database. The major disadvantage of RFID technology when used outdoors is its short range [76]. Active transponders are connected to a battery and transmit radio waves actively, which gives them a wider range than passive ones (about 100 m, compared to 3 m or less). In a review [8], technologies to quantify beef cattle behaviours in U.S. beef production systems were presented, including accelerometers, radio frequencies and global positioning systems. In conclusion, each behaviour monitoring system has the potential to enhance research on animal welfare, health, nutrition and reproduction. However, the commercial implementation of remote disease identification technologies hinges upon several economically beneficial outcomes, such as their potential to reduce labour costs and animal mortality and increase performance.

### 5.2. Networks

Many different wireless technologies have been applied for diverse purposes in agriculture, depending on the economic, accessibility and capability factors [77]. The specifications of wireless communication technologies implemented in the IoT in an agricultural context have been presented by several authors [78,79,80]. Wireless Sensor Networks (WSN) consist of a transceiver, sensors, microcontrollers and energy sources. The network is formed by sensor nodes with the capability of data storage and processing and which, unlike RFID systems, can communicate wirelessly with each other [76,81,82]. WSN are often used when there is a need to monitor physical environmental conditions remotely. Applications with sensor networks to identify and track objects have increased in importance in recent years, partly due to the development of small and cheap multifunctional sensor nodes with low energy consumptions. The focus is on developing sufficiently technically reliable systems, while studies that evaluate how well the systems work for the assessment of animal welfare are largely lacking. As far as the monitoring of grazing livestock is concerned, studies have been made to evaluate the input sensor components for variations in the time and resolution in space [82].

Molapo et al. [83] presented a new, inexpensive and relatively simple WSN system to track livestock and monitor their activity in real time using Wi-Fi. Tags recorded the movements of the animals using accelerometers, and the geographical location was calculated through trilateration with information from nearby lighthouses. Information about the animals’ identity, location and activity were sent to a base station node, which passed that on for storage on a web server. The functionality of the system, in terms of range, position accuracy and ability to detect and store information, was evaluated by moving around the tags by hand. With a clear view, an area of 400 m^2^ could be monitored without any information being lost, and 2D positions (movements on flat ground) could be estimated with a position accuracy of less than 3 m.

Jukan et al. [53] found that the richness and heterogeneity of wireless technologies used for animal tracking reflect the diversity of animal species and conducted a systematic review of smart computing and sensing technologies for animal welfare purposes. The authors found numerous descriptions of hybrid wireless networks, in which a mobile node has connectivity with an infrastructure network such as the Internet or a separate local network, and they identified many technical challenges with respect to the bandwidth and capacity management in the integration of video- and camera-based wireless sensor networks. Special attention needs to be paid to the robustness and adaptability of the system to reduce the maintenance required in remote settings, and some settings will need to be ultra-low-power and low-cost.

For livestock, a standard-based integration with wireless 3G networks and smartphone-based applications is a common approach [53]. Most of the sensor systems reported support network connectivity, but only some of the sensor systems reviewed connect to a common shared infrastructure, like the cloud, which is required to be able to share data and the best practices. The key challenges for a wider adoption of commercial smart farming services are trade-offs between the cost, battery power and network connectivity in practical livestock monitoring scenarios [53].

Surprisingly, Jukan et al. [53] did not to find any research in the emerging area of 5G cellular networks with a focus on livestock or networks for farm animal welfare. However, in several technologically well-developed countries, broadband in the countryside is still absent, which means that not even the advantages of 3 or 4G can be fully utilised. Around 50% of rural households in the EU reported in 2017 that they lacked broadband connectivity due to difficulties with terrain and the costs of expanding the cable network [84].

### 5.3. GPS Monitoring

In the last 20–25 years, techniques for monitoring habitat selections and the movements of wild animals [85,86,87] and free-ranging livestock [88] have been explored by equipping the animals with collars with a positioning receiver. The Global Positioning System (GPS) relies on radio signals from specialised satellites available at the given time of the positioning. The animal locations are often combined with relevant environmental information, such as vegetation type, topography, proximity to water and distance to human activities and infrastructures, to evaluate the habitat selection of animals [89,90]. Information on habitat selection is commonly used in assessments of animal habitats [91,92,93,94,95]. An analysis of the length of movement paths between the recorded animal locations may reveal the circadian rhythm of an animal and can show whether the movement patterns are regular or change over time [96,97,98]. For example, it may be possible to determine where an animal stops to graze if locations are recorded at least every five minutes [98,99]. It is also possible to estimate calving sites based on two-hour data or even coarser data [100,101] and determine when parturition starts [102] and whether the offspring survives the first few days after birth [103]. Such information can be used to evaluate calving sites in relation to environmental factors and estimate the number of calves born and, at a later stage, calf survival.

GPS technology also facilitates the study of interactions between animals [104] to help determine if a pasture is overused or not [82] and how access to high- or low-quality pastures affects animals’ group dynamics. Lean pastures seem to split animals into smaller groups [105]. Using GPS collars, pedometers and heart rate sensors to compare Beefmaster-Simford crosses and Baladi cattle, Aharoni et al. [106] found the Baladi cattle to be more active throughout the year and to graze more than the Beefmaster-Simfords. Animals of both breeds generally moved less during the vegetative season compared to the nonvegetative season. Beker et al. [107] used similar techniques estimating the correlation between energy consumption and time of movement of sheep and goats. Moreover, studies of interactions between predator and prey animals may give important information on how to manage livestock and free-ranging domesticated animals in a predator-rich environment [108,109,110,111]. For example, it has been suggested [110] that there is no reason to increase the surveillance of cattle herds, as there was little risk that they would select the same habitat as brown bears. In another study, using a combination of RFID and GPS collars equipped with a sensor to recognise the RFID collar within a defined distance (proximity sensor), it was possible to track the brown bear predation of reindeer calves [111,112]. The use of RFID proximity collars was shown to be an efficient way to monitor a large group of animals at a reasonable cost. All female reindeer (at least 900 females in each of the two herds) were provided with RFID collars, and the brown bears were provided with GPS collars. When a reindeer female came within 100 m of a GPS bear, the GPS receiver started recording their positions at one-min intervals, which made it possible to identify locations where the bear stopped to kill a reindeer calf and, thus, estimated the number of calves killed by a brown bear. This provided information on how to manage the brown bear population in the region [112].

The combination of GPS with 3D accelerometers or cameras has also proven successful, as it increases the possibility of recording detailed behaviours [86,98,106,107]. Monitoring sheep and cattle in New Zealand using sensors that logged animal positions and urine volumes, Betteridge et al. [113] studied the correlation between grazing and urination. This information could then be used when applying nitrogen-reducing products to pastures. Virgilio et al. [114] studied three Merino sheep in Patagonia, Chile equipped with head-mounted 3D activity meters, as well as 2D activity meters and GPS receivers, around their necks for 15 days. The activity sensors recorded 40 measurements per second and the GPS receiver one position per minute. Validating the 3D and 2D sensors with behavioural observations, they, for example, showed that the sensors managed to record the bite rates of the sheep with an almost perfect fit, which provided an opportunity to evaluate the animals’ ability to find and feed on high-quality forage patches. All bites observed were detected by the 3D sensors on the sheep’s heads, and 97% of the bites were detected by the 2D sensor attached to the collar.

Together, the mentioned GPS studies showed that purely positional information about a grazing animal provides important clues to assess its welfare. However, combining GPS with other sensors that provide more detailed information about the animals’ condition or behaviour can give additional essential information to evaluate the welfare of free-ranging animals in pastures more accurately.

### 5.4. Position Accuracy

There are two types of errors associated with GPS: unsuccessful fixed acquisitions result in missing location data, while location inaccuracies result in incorrect data [115,116]. The more often a position is determined, the better the ability to register a position (the fixed acquisition rate), while the position accuracy does not appear to be affected [117]. The position accuracy, which refers to the measurement error or precision of the estimated location, also varies with the type of GPS collar used [117] and the number and coverage of the satellites at the time of positioning. Recent studies have shown that a device’s ability to accurately define a location (positioning measurement error or precision) may vary between 43% and 99%, and that there is a variation in the precision both during the day and throughout the year [117]. With a good scattering of GPS satellites in the sky, positioning becomes safer compared to if the satellites happened to be close to each other. The providers of GPS equipment usually specify a value (Dilution of Precision, DOP) that indicates the uncertainty of each position, depending on the number of satellites used and their distribution in the sky [117]. The DOP value can help evaluate the precision of a position and allows for discarding particularly uncertain positions. When an animal’s altitude varies greatly, the DOP value might be misleading [118].

Apart from using measures such as the DOP value, the reliability of GPS equipment can be tested using stationary receivers deployed in the terrain 1–1.5 m aboveground (to simulate the height of the animals) and set to record positions in two- to five-min intervals [117]. From such tests, it is known that the measurement error increases with the coverage and density of forest vegetation. The error is 19–30 m in a dense forest with more than 70% coverage, 16 m in a sparse forest with 41–70% coverage and 7–13 m in a sparse forest with 0–40% coverage [117]. The topography can also interfere with the signals, and with less than 30% available sky, the measurement error has been estimated at 10–13 m [119]. Tests of GPS collars show that the measurement error can be reduced by 5–33% when receivers are in motion compared to stationary use. In addition, the position quality may be increased if GPS receivers are combined with accelerometers; the drift and direction of the animal recorded with the accelerometer can be used in dead reckoning to estimate the animal’s path [114,120]. In this way, fewer GPS positions need to be determined, and the battery life can be extended.

## 6. Technologies to Control Animal Movements Outdoors

### 6.1. Moving Animals with Drones

Herding animals with drones seems to be increasingly common, but there are only a few scientific reports. Recently, McDonnell and Torcivia [121] reported using drones instead of helicopters to move feral horses into a trap. Other sources of information include reindeer that were pushed forward with drones in the same way as with a helicopter, i.e., the herd was pushed forward at a reasonable distance and speed (pers. comm., M. Kuhmunen, Jokkmokk, Sweden, 29 January 2019). There are also media reports on the use of drones to herd sheep [122], as well as cattle [123].

Drones are helpful if the animals end up in areas that might be dangerous for the herder to travel—for example, on water reservoirs with unsafe ice or in rough and steep terrain—and as a cost-effective alternative to helicopters. There are few studies exploring the response of herded animals to the presence (sight and sound) of flying drones. Brunberg et al. [124] used a drone to simulate a predator in a flock of sheep and found a greater unease among the sheep when exposed to the drone compared to the more familiar sheep dogs or humans. The sheep were, in their natural environment, exposed to birds of prey, which may explain the response.

### 6.2. Virtual Fences

A virtual fence serves as an enclosure or border but without any physical barrier [125,126]. The fence is, hence, invisible. Instead of facing a visible barrier, possibly accompanied by an electric shock if the barrier is touched, the animal has to learn to associate a sound signal with an electric shock and to turn around or stop to avoid the shock. Virtual fences can decrease the workload of the livestock keeper and provide a more flexible fencing system. There are few commercially available virtual fences, and they all consist of a collar-mounted device attached to each animal and software to define the position of the fence. When the animal approaches the virtual fence, the collar emits a warning signal—usually, a sound. If the animal continues ahead and is about to cross the fence line, a painful but harmless electric shock is elicited. The strength of the shock varies between different commercial systems and studies.

The position of the virtual border can be defined by coordinates and GPS signals or by a ground cable that communicates with the collars. GPS-based fences are superior in reducing the stockperson’s workload, since the border can be moved using a mobile application. However, an advantage with a visible ground cable is that it gives the animals a visual cue in addition to the sound, which may facilitate learning and understanding [127]. A few studies have tested fences with other warning signals than sound [128], no warning signals at all [129] or other deterrents than electric shocks [130]. These studies indicate that cattle may remember and avoid the location of a virtual fence with no preceding warning signal [128,129] and that irritating sounds are not as effective as electric shocks from a conventional fence, although they do influence cattle movements [130].

Cattle [127,131,132,133,134], sheep [135,136,137,138,139] and goats [140,141] have been tested in different virtual fencing systems. Due to differences in animal species, animal group sizes, fencing types, enclosure sizes and animal training, the studies are difficult to compare. For example, the sizes of virtual enclosures varied from a single 6-m broad virtual border separating the animals from a feed attractant [128] to pastures of several hectares divided with a single virtual fence line [134]. In addition, some of the studies aimed to investigate the practical functioning of the system without a clear focus on animal welfare. Some of the researchers did not report the number of electric shocks that the animals received, which is important for evaluating animal welfare effects [127,135].

Research shows that livestock, in most cases, can learn to associate the sound signal with the electric shock [131,132,133,134,135,136] and that they, hence, after a learning period, at least in some cases, will stay on one side of the virtual fence. Campbell et al. [134] kept ten heifers in a virtual enclosure over ten days, and the animals remained in the enclosure, with a few exceptions. Marini et al. [136] were able to keep a group of sheep in a virtual enclosure for five days. However, several studies report that some of the animals pass the virtual border (e.g., [131,137,138,139,142]. For example, Brunberg et al. [139] tested a GPS-based virtual fencing system on small groups of sheep wearing collars and their young lambs who had no collars and, thus, could move freely over the border. The authors found that the sheep spent almost half of the time outside the enclosure.

There are also large individual differences in how quickly the animals adapt to the system [142]. Campbell et al. [133] found that the studied heifers received 3–23 electric shocks each during a series of eight experimental tests. The ewes studied by Brunberg et al. [139] could receive a maximum of five shocks per day, and the total number of shocks per animal was 6–20 over four days.

## 7. Animal Welfare Risks When Using Digital Technology

There are some obvious animal welfare risks when using digital technology to monitor livestock. There are general risks for all types of sensors and computer technologies in the monitoring of animal welfare that relate to technical functions, connectivity and energy, as well as the performance of a system to capture important expressions of animal welfare. If any of these parts fail, and there is too much confidence in the functions and information produced by the PLF systems, the animals’ welfare can be at risk. Risks exist both outdoors and indoors, while others only apply to outdoors or to large pastures. Animals equipped with sensors or GPS collars can get bruises and abrasive ulcers from the equipment. The risk increases when sensors are used on growing animals where, for example, the size of the collar has to be adjusted gradually [37,143,144]. When collars are used on sheep, the wool can affect the fit, which is especially important to consider in virtual fencing systems where electrodes need to be in contact with the skin [135,137,138]. Collars and devices can get stuck in vegetation and cause injury, stress and possibly even death. Animals may also react negatively to the application of devices to their body. In an experiment with tail-mounted calving sensors [145], 80% of farmers stated that the animals reacted negatively when the sensor was attached to the tail root, and 20% observed so much damage that amputation of the tail was necessary. No information on the animal welfare effects of the use of rumen boluses has been found.

The incorrect use of drones for herding is likely to cause negative stress effects, but scientific studies are scarce. Wiklund and Malmfors [146] did not find the helicopter herding of reindeer to affect the muscle glycogen content, ultimate meat pH, blood metabolites or the frequency of abomasal lesions. However, the continuous use of drones for herding would require more studies regarding the effects on animal welfare.

Some animals may lack adaptation to or acceptance of virtual fencing systems, likely causing stress. Most studies indicate that there are large individual differences in learning capacities and behavioural responses, which may be a welfare problem for slow learners who will receive many electric shocks. There is no research on the significance of different learning mechanisms and the long-term effects of virtual fencing. Information on the number of electric shocks is often missing [127,135], which makes it difficult to evaluate the possible effects on animal welfare. Lee et al. [131] compared the behaviours and some physiological parameters in cattle that were either exposed to three electric shocks or kept in chutes with their heads fixed and concluded that the electric shocks and head fixation were equally stressful. Kearton et al. [147] studied differences in the behaviours, temperatures and cortisol levels in sheep that were exposed to a disturbing sound signal, dog bark, restraint or an electric shock and found that the electric shock was less stressful than the restraint but more than the dog bark and noise. McDonald et al. [148] reported that cattle enclosed with a physical electric fence touched the fence between zero and three times; 84% of the animals did not interact at all or only once. Another group of animals touched the fence on average twice during the first half-day, while only one of the animals received one electric shock during the remaining seven days. This indicates that more electric shocks are received and that the individual variations are larger using a virtual fence compared to a physical electric fence. This may result in a higher stress level compared to physical fences, especially during the learning phase.

Technical issues with virtual fences have been reported [132,139] that may also have animal welfare consequences. Unlike some physical fences, virtual fences do not protect from predator attacks, which is a clear disadvantage in areas with predators. On the other hand, virtually fenced animals can probably escape if frightened or attacked by predators. If passers-by, with or without the company of a dog, are not aware of a virtual fence, they may inadvertently cross the border and come into contact with the enclosed animals, thus frightening or even harming them.

If stockpersons rely too much on a digital monitoring system, there is an obvious risk of serious animal welfare consequences if the system ceases to function. Care measures might not be taken in time or not at all. The lack of regular positive human contacts with the animals can also put them under greater stress when handled. Therefore, these systems can never completely replace manual inspections.

## 8. Conclusions

The automated sensor-based monitoring of cattle and sheep in large pastures offers great potential for improving animal welfare, as it has the capacity to capture animals’ physiology and behaviour, as well as environmental factors, in real time and more or less continuously. To some extent, but not completely, it can replace manual labour, thus saving time and money. The electronic positioning and data transfer systems include Radio Frequency Identification (RFID), Wireless Sensor Networks (WSN), Global Positioning (GPS), Wireless Sensor Networks (WSN), the Internet of Things (IoT) and Low-Power Wide-Area (LPWA) solutions. Stationary or drone-mounted cameras can locate and count animals, as well as herd them. Digitally defined virtual fences can keep animals within a predefined area without the use of physical barriers, relying on acoustic signals and weak electric shocks.

## 9. Suggested Future Research

Further research on remote monitoring and the management of livestock in large pastures is motivated by the need for farmers to more easily find and manage animals that need care and treatment, which reduces the loss of animals and has the potential to increase animal welfare. The benefits include a reduction in the time that must be spent finding animals in large pastures or setting up fences and managing pastures. It is important to find efficient and reliable technical solutions. Sensors in wearabl devices with a server connection to the “cloud” provide great opportunities but need to be further explored. The possibilities of saving battery power by reducing signal sampling and connection intervals without compromising sensitivity and specificity need to be studied. New imaging technology provides the opportunity to use wearable cameras to detect animals with impaired health or welfare. This opens up the opportunity for more research and the development of drone technology. When it comes to virtual fencing, the primary goal of future research must be to ensure animal welfare.

## Figures and Tables

**Table 1:** Examples of the sensors used in dairy farming, what they measure and what the alarm signal can inform the stockperson [14].

Type of Sensor	Measurement	Information
Activity	Activity, rumination, lying time, step count	Oestrus, calving, lameness, general health
pH sensor	Rumen pH	Rumen acidosis
Camera	Activity, feed intake, body shape	Ketosis, body condition, lameness, mastitis
Thermometer, thermography	Body temperature thermal body surface radiation	Water intake, calving, infection, lameness, general health
Microphone	Rumination time	Rumen function, general health, oestrus, calving

**Table 2:** Commercially available and scientifically validated animal-based sensors for monitoring ruminant behaviours in pastures. ^1^ *ρ* = Spearman’s correlation coefficient, *r* = Pearson’s correlation coefficient, CCC = concordance correlation coefficient, Se = sensitivity, Sp = specificity, PPV = positive predictive value and *r*^2^ = coefficient of determination.

Product	Manufacturer	Type & Features	Technology	Recorded Behaviour	Performance ^1^	References
RumiWatch System	Itin + Hoch (Liestal, Switzerland)	Nose: Eating time, Rumination Leg: Activity	Accelerometer Pressure gauge	Eating time Rumination Lying time Movement Standing time	*ρ* 0.91–0.98; CCC 0.95–0.98 *ρ* 0.97–0.98; CCC 0.93–0.94 *ρ* 0.99; CCC 1.00 *ρ* 0.78; CCC 0.92 *ρ* 0.97; CCC 1.00	[47,48,49]
IceTag	IceRobotics (Edinburgh, UK)	Leg: Activity	Accelerometer	Lying time Step count Standing time	Se 0.99 *r*^2^ 0.97 Se 0.98	[50]
CowManager SensOor	Agis Automatisering (Harmelen, The Netherlands)	Ear: Activity, Eating time, Rumination	Accelerometer	Activity Rumination Eating time	CCC 0.19–0.52; *r* 0.20–0.65 CCC 0.71; *r* 0.72 CCC 0.88; *r* 0.88	[51]
Heatime HR LD System	SCR Dairy (Netanya, Israel)	Neck: Activity, Eating time, Rumination	Accelerometer	Activity Eating time Rumination	CCC 0.95; *r* 0.97; Se 0.77; Sp 0.99; PPV 0.93 CCC 0.99; *r* 0.99; Se 0.98; Sp 0.97; PPV 0.99 CCC 0.80; *r* 0.80; Se 0.87; Sp 0.98; PPV 0.91	[52]

## Data Availability

Not applicable.

## References

[1] Stampa E., Schipmann-Schwarze C., Hamm U. (2020). Consumer perceptions, preferences, and behavior regarding pasture-raised livestock products: A review. Food Qual. Prefer..

[2] Rook A.J., Tallowin J.R.B. (2003). Pasture Ecology Meeting Plant-Herbivore Relationships. Anim. Res..

[3] Yu H., Li Y., Oshunsanya S.O., Are K.S., Geng Y., Saggar S., Liu W. (2019). Re-introduction of light grazing reduces soil erosion and soil respiration in a converted grassland on the Loess Plateau, China. Agric. Ecosyst. Environ..

[4] Teague W.R., Apfelbaum S., Lal R., Kreuter U.P., Rowntree J., Davies C.A., Conser R., Rasmussen M., Hatfield J., Wang T. (2016). The role of ruminants in reducing agriculture’s carbon footprint in North America. J. Soil Water Conserv..

[5] Lovarelli D., Bacenetti J., Guarino M. (2020). A review on dairy cattle farming: Is precision livestock farming the compromise for an environmental, economic and social sustainable production?. J. Clean. Prod..

[6] Wathes C.M., Kristensen H.H., Aerts J.-M., Berckmans D. (2008). Is precision livestock farming an engineer’s daydream or nightmare, an animal’s friend or foe, and a farmer’s panacea or pitfall?. Comput. Electron. Agric..

[7] Berckmans D. (2017). General introduction to precision livestock farming. Anim. Front..

[8] Richeson J.T., Lawrence T.E., White B.J. (2018). Using advanced technologies to quantify beef cattle behaviour. Transl. Anim. Sci..

[9] Fawcett T. (2006). An introduction to ROC analysis. Pattern. Recogn. Lett..

[10] Bouchon M., Bach A., Meunier B., Ternman E., Van Reenen K., Veissier I., Munksgaard L. (2019). A Checklist to Validate Sensor Output for the Recording of Cattle Behaviour. http://www.smartcow.eu/wp-content/uploads/2019/06/D7.1-Guidelines-for-validation-of-sensor-output.pdf.

[11] International Committee for Animal Recording Sensor Devices Task Force. 2018, Rome, Italy. https://www.icar.org/index.php/technical-bodies/task-forces/sensor-devices-task-force-landing-page/#.

[12] Helwatkar A., Riodran M., Walsh J. (2014). Sensor Technology for Animal Health Monitoring. Int. J. Smart Sens. Intell. Syst..

[13] Rutten C.J., Velthuis A.G.J., Steeneveld W., Hogeveen H. (2013). Invited review: Sensors to support health management on dairy farms. J. Dairy Sci..

[14] Palczynski L. (2019). Third Annual Report for Researchers on Research Priorities on the Use of Sensor Technologies to Improve Productivity and Sustainability on Dairy Farms. https://www.4d4f.eu/sites/default/files/4D4F%20Dairy%20Sensor%20Research_2019_0.pdf.

[15] McCorkell R., Wynne-Edwards K., Windeyer C., Schaefer A. (2014). Limited efficacy of Fever Tag(^®^) temperature sensing ear tags in calves with naturally occurring bovine respiratory disease or induced bovine viral diarrhea virus infection. Can. Vet. J..

[16] Koltes J.E., Koltes D.A., Mote B.E., Tucker J., Hubbell D.S. (2018). Automated collection of heat stress data in livestock: New technologies and opportunities. Transl. Anim. Sci..

[17] Humer E., Ghareeb K., Harder H., Mickdam E., Khol-Parisini A., Zebeli Q. (2015). Peripartal changes in reticuloruminal pH and temperature in dairy cows differing in the susceptibility to subacute rumen acidosis. J. Dairy Sci..

[18] Gasteiner J., Guggenberger T., Häusler J., Steinwidder A. (2012). Continuous and Long- Term Measurement of Reticuloruminal pH in Grazing Dairy Cows by an Indwelling and Wireless Data Transmitting Unit. Vet. Med. Int..

[19] Antanaitis R., Žilaitis V., Juozaitiene V., Stoškus R. (2016). Usefulness of Acidity and Temperature of the Rumen and Abomasum in Diagnosing SARA in Dairy Cows after Calving. Pol. J. Vet. Sci..

[20] Jonsson N., Kleen J.L., Wallace R.J., Andonovic I., Michie C., Farish M., Mitchell M., Duthie C.A., Jensen D.B., Denwood M.J. (2019). Evaluation of reticuloruminal pH measurements from individual cattle: Sampling strategies for the assessment of herd status. Vet. J..

[21] Fuchs B., Sørheim K.M., Chincarini M., Brunberg E., Stubsjøen S.M., Bratbergsengen K., Hvasshovd S.O., Zimmermann B., Støbet Lande U., Grøva L. (2019). Heart rate sensor validation and seasonal and diurnal variation of body temperature and heart rate in domestic sheep. Vet. Anim. Sci..

[22] Weary D.M., Huzzey J.M., Von Keyserlingk M.A.G. (2009). Board-invited Review: Using behaviour to predict and identify ill health in animals. J. Anim. Sci..

[23] Rutter M. Current and future prospects for the automatic recording and control of ruminant foraging on farms. Proceedings of the Third Dairy Care Conference 2015.

[24] Delagarde R., Lamberton P. (2015). Daily grazing time of dairy cows is recorded accurately using the Lifecorder Plus device. Appl. Anim. Behav. Sci..

[25] Chelotti J.O., Vanrell S.R., Milone D.H., Utsumi S.A., Galli J.R., Rufiner H.L., Giovanini L.L. (2016). A real-time algorithm for acoustic monitoring of ingestive behaviour of grazing cattle. Comp. Electron Agric..

[26] Ruuska S., Kajava S., Mughal M., Zehner N., Mononen J. (2016). Validation of a pressure sensor-based system for measuring eating, rumination and drinking behaviour of dairy cattle. Appl. Anim. Behav. Sci..

[27] Andriamandroso A.L.H., Lebeau F., Beckers Y., Froidmont E., Dufrasne I., Heinesch B., Dumortier P., Blanchy G., Blaise Y., Bindelle J. (2017). Development of an open-source algorithm based on inertial measurement units (IMU) of a smartphone to detect cattle grass intake and ruminating behaviours. Comp. Electron Agric..

[28] Mansbridge N., Mitsch J., Bollard N., Ellis K., Miguel-Pacheco G.G., Dottorini T., Kaler J. (2018). Feature Selection and Comparison of Machine Learning Algorithms in Classification of Grazing and Rumination. Behaviour in Sheep. Sensors.

[29] Lalaina A., Andriamandroso H., Bindelle J. (2016). A review on the use of sensors to monitor cattle jaw movements and behaviour when grazing. Biotechnol. Agron. Soc. Environ..

[30] Duncan N.B., Meyer A.M. (2019). Locomotion behaviour changes in peripartum beef cows and heifers. J. Anim. Sci..

[31] Cyples J.A., Fitzpatrick C.E., Leslie K.E., DeVries T.J., Haley D.B., Chapinal N. (2012). Short communication: The effects of experimentally induced Escherichia coli clinical mastitis on lying behaviour of dairy cows. J. Dairy Sci..

[32] Medrano-Galarza C., Gibbons J., Wagner S., de Passillé A.M., Rushen J. (2012). Behavioural changes in dairy cows with mastitis. J. Dairy Sci..

[33] Fogsgaard K.K., Bennedsgaard T.W., Herskin M.S. (2015). Behavioural changes in freestall-housed dairy cows with naturally occurring clinical mastitis. J. Dairy Sci..

[34] Neave H.W., Lomb J., Weary D.M., LeBlanc S.J., Huzzey J.M., von Keyserlingk M.A.G. (2018). Behavioral changes before metritis diagnosis in dairy cows. J. Dairy Sci..

[35] Itle A.J., Huzzey J.M., Weary D.M., von Keyserlingk M.A.G. (2015). Clinical ketosis and standing behaviour in transition cows. J. Dairy Sci..

[36] Chapinal N., de Passillé A.M., Rushen J., Wagner S. (2010). Automated methods for detecting lameness and measuring analgesia in dairy cattle. J. Dairy Sci..

[37] Kokin E., Praks J., Veermäe I., Poikalainen V., Vallas M. (2014). IceTag3DTM accelerometric device in cattle lameness detection. Agron. Res..

[38] Thorup V.M., Munksgaard L., Robert P., Erhard H.W., Thomsen P.T., Friggens N.C. (2015). Lameness detection via leg-mounted accelerometers on dairy cows on four commercial farms. Animal.

[39] Barwick J., Lamb D., Dobos R., Schneider D., Welch M., Trotter M. (2018). Predicting lameness in sheep activity using tri-axial acceleration signals. Animals.

[40] Marchesini G., Mottaran D., Contiero B., Schiavon E., Segato S., Garbin E., Tenti S., Andrighetto I. (2018). Use of rumination and activity data as health status and performance indicators in beef cattle during the early fattening period. Vet. J..

[41] Forbes A.B., Huckle C.A., Gibb M.J., Rook A.J., Nuthall R. (2000). Evaluation of the effects of nematode parasitism on grazing behaviour, herbage intake and growth in young grazing cattle. Vet. Parasitol..

[42] Forbes A.B., Huckle C.A., Gibb M.J. (2004). Impact of eprinomectin on grazing behaviour and performance in dairy cattle with sub-clinical gastrointestinal nematode infections under continuous stocking management. Vet. Parasitol..

[43] Szyszka O., Tolkamp B.J., Edwards S.A., Kyriazakis I. (2013). Do the changes in the behaviours of cattle during parasitism with Ostertagia ostertagi have a potential diagnostic value?. Vet. Parasitol..

[44] Burgunder J., Petrželková K.J., Modrý D., Kato A., MacIntosh A.J.J. (2018). Fractal measures in activity patterns: Do gastrointestinal parasites affect the complexity of sheep behaviour?. Appl. Anim. Behav. Sci..

[45] Högberg N., Lidfors L., Hessle A., Arvidsson Segerkvist K., Herlin A., Höglund J. (2019). Effects of nematode parasitism on activity patterns in first-season grazing cattle. Vet. Parasitol..

[46] Sepúlveda-Varas P., Proudfoot K.L., Weary D.M., von Keyserlingk M.A.G. (2016). Changes in behaviour of dairy cows with clinical mastitis. Appl. Anim. Behav. Sci..

[47] Werner J., Leso L., Umstätter C., Niederhauser J., Kennedy E., Geoghegan A., Shalloo L., Schick M., Brien B.O. (2018). Evaluation of the RumiWatchSystem for measuring grazing behaviour of cows. J. Neurosci. Methods.

[48] Werner J., Viel J., Niederhauser J., O’Leary N., Umstätter C., O’Brien B. Validation of new algorithms for the RumiWatch noseband sensor to detect grazing behaviour of dairy cows. Sustainable Meat and Milk Production from Grasslands. Proceedings of the 27th General Meeting of the European Grassland Federation.

[49] Rombach M., Münger A., Niederhauser J., Südekum K., Schori F. (2018). Evaluation and validation of an automatic jaw movement recorder (RumiWatch) for ingestive and rumination behaviours of dairy cows during grazing and supplementation. J. Dairy Sci..

[50] Ungar E.D., Nevo Y., Baram H., Arieli A. (2018). Evaluation of the IceTag leg sensor and its derivative models to predict behaviour, using beef cattle on rangeland. J. Neurosci. Methods.

[51] Pereira G.M., Heins B.J., Endres M.I. (2018). Validation of an ear-tag accelerometer sensor to determine rumination, eating, and activity behaviours of grazing dairy cattle. J. Dairy Sci..

[52] Molfino J., Clark C.E.F., Kerrisk K.L., Garciá S.C. (2017). Evaluation of an activity and rumination monitor in dairy cattle grazing two types of forages. Anim. Prod. Sci..

[53] Jukan A., Masip-Bruin X., Amla N. (2017). Smart computing and sensing technologies for animal welfare: A systematic review. ACM Comput. Surv..

[54] Norouzzadeh M.S., Nguyen A., Kosmala M., Swanson A., Palmer M.S., Packer C., Clune J. (2018). Automatically Identifying, Counting, and Describing Wild Animals in Camera-trap Images with Deep Learning. Proc. Natl. Acad. Sci. USA.

[55] Guzhva O., Nilsson M., Ardö H., Herlin A.H., Åström K., Bergsten C. (2016). Feasibility study for the implementation of an automatic system for the detection of social interactions in the waiting area of automatic milking stations by using a video surveillance system. Comp. Electron. Agric..

[56] Ren K., Karlsson J., Liuska M., Hartikainen M., Hansen I., Jørgensen G.H.M. (2020). A sensor-fusion-system for tracking sheep location and behaviour. Int. J. Distrib. Sens. N..

[57] Porto S.M.C., Arcidiacono C., Giummarra A., Anguzza U., Cascone G. (2014). Localisation and identification performances of a real-time location system based on ultra wide band technology for monitoring and tracking dairy cow behaviour in a semi-open free-stall barn. Comp. Electron. Agric..

[58] Mulero-Pázmány M., Barasona J.A., Acevedo P., Vicente J., Negro J.J. (2015). Unmanned Aircraft Systems complement biologging in spatial ecology studies. Ecol. Evol..

[59] Christie K.S., Gilbert S.L., Brown C.L., Hatfield M., Hanson L. (2016). Unmanned aircraft systems in wildlife research: Current and future applications of a transformative technology. Front. Ecol. Environ..

[60] Schroeder N.M., Panebianco A., Gonzalez Musso R., Carmanchahi P. (2020). An experimental approach to evaluate the potential of drones in terrestrial mammal research: A gregarious ungulate as a study model. R. Soc. Open Sci..

[61] Rümmler M.-C., Mustafa O., Maercker J., Peter H.-U., Esefeld J. (2018). Sensitivity of Adélie and Gentoo penguins to various fight activities of a micro UAV. Polar Biol..

[62] Ditmer M.A., Werden L.K., Tanner J.C., Vincent J.B., Callahan P., Iaizzo P.A., Laske T.G., Garshelis D.L. (2019). Bears habituate to the repeated exposure of a novel stimulus, unmanned aircraft systems. Conserv. Phys..

[63] Eikelboom J.A.J., Wind J., van de Ven E., Kenana L.M., Schroder B., de Knegt H.J., van Langevelde F., Prins H.H.T. (2019). Improving the precision and accuracy of animal population estimates with aerial image object detection. Methods Ecol. Evol..

[64] Kellenberger B., Marcos D., Tuia D. (2018). Detecting mammals in UAV images: Best practices to address a substantially imbalanced dataset with deep learning. Remote Sens. Environ..

[65] Torney C.J., Lamont M., Debell L., Angohiatok R.J., Leclerc L.-M., Berdahl A.M. (2018). Inferring the rules of social interaction in migrating caribou. Philos. Trans. R. Soc. Lond. B Biol. Sci..

[66] Ringhofer M., Go C.K., Inoue S.S., Mendonça R., Hirata S., Kubo T., Ikeda K., Yamamoto S. (2020). Herding mechanisms to maintain the cohesion of a harem group: Two interaction phases during herding. J. Ethol..

[67] Mulero-Pázmány M., Stolper R., Van Essen L.D., Negro J.J., Sassen T. (2014). Remotely piloted aircraft systems as a rhinoceros anti-poaching tool in Africa. PLoS ONE.

[68] Corcoran E., Denman S., Hanger J., Wilson B., Hamilton G. (2019). Automated detection of koalas using low-level aerial surveillance and machine learning. Sci. Rep..

[69] Andrew W., Greatwood C., Burghardt T. Visual localisation and individual identification of Holstein Friesian cattle via deep learning. Proceedings of the IEEE International Conference on Computer Vision, (ICCVW).

[70] Xu B., Wang W., Falzon G., Kwan P., Guo L., Sun Z., Li C. (2020). Livestock classification and counting in quadcopter aerial images using Mask R-CNN. Int. J. Remote Sens..

[71] He K., Gkioxari G., Dollár P., Girshick R. Mask R-CNN. Proceedings of the 2017 IEEE International Conference on Computer Vision (ICCV).

[72] Danish M. (2018). Beef Cattle Instance Segmentation Using Mask R-Convolutional Neural Network. Master’s Thesis.

[73] Xu B., Wang W., Falzon G., Kwan P., Guo L., Chen G., Tait A., Schneider D. (2020). Automated cattle counting using Mask R-CNN in quadcopter vision system. Comput. Electron. Agric..

[74] Schwartzkopf-Genswein K.S., Huisma C., McAllister T.A. (1999). Validation of a radio frequency identification system for monitoring the feeding patterns of feedlot cattle. Livest. Prod. Sci..

[75] Voulodimos A.S., Patrikakis C.Z., Sideridis A.B., Ntafis V.A., Xylouri E.M. (2010). A complete farm management system based on animal identification using RFID technology. Comput. Electron. Agric..

[76] Ruiz-Garcia L., Lunadei L., Barreiro P., Robla I. (2009). A review of wireless sensor technologies and applications in agriculture and food industry: State of the art and current trends. Sensors.

[77] Villa-Henriksen A., Edwards G.T., Pesonen L.A., Green O., Sørensen C.A.G. (2020). Internet of Things in arable farming: Implementation, applications, challenges and potential. Biosyst. Eng..

[78] Jawad H., Nordin R., Gharghan S., Jawad A., Ismail M. (2017). Energy-efficient wireless sensor networks for precision agriculture: A review. Sensors.

[79] Ray P.P. (2017). Internet of Things for smart agriculture: Technologies, practices and future direction. J. Ambient Intell. Smart Environ..

[80] Tzounis A., Katsoulas N., Bartzanas T., Kittas C. (2017). Internet of Things in agriculture, recent advances and future challenges. Biosyst. Eng..

[81] Akyildiz I.F., Ian F., Su W., Sankarasubramaniam Y., Cayirci E. (2002). A survey on sensor networks. IEEE Commun. Mag..

[82] Handcock R.N., Swain D.L., Bishop-Hurley G.J., Patison K.P., Wark T., Valencia P., Corke P., O’Neill C.J. (2009). Monitoring Animal Behaviour and Environmental Interactions Using Wireless Sensor Networks, GPS Collars and Satellite Remote Sensing. Sensors.

[83] Molapo N.A., Malekian R., Nair L. (2019). Real-Time Livestock Tracking System with Integration of Sensors and Beacon Navigation. Wirel. Pers. Commun..

[84] European Commission (2018). Broadband Coverage in Europe 2017. European Commission DG Communications Networks, Content & Technology.

[85] Kays R., Crofoot M.C., Jetz W., Wikelski M. (2015). Terrestrial animal tracking as an eye on life and planet. Science.

[86] Wilmers C.C., Nickel B., Bryce C.M., Smith J.A., Wheat R.E., Yovovich V. (2015). The golden age of bio-logging: How animal-borne sensors are advancing the frontiers of ecology. Ecology.

[87] Tucker M.A., Böhning-Gaese K., Fagan W.F., Fryxell J.M., Van Moorter B., Alberts S.C., Ali A.H., Allen A.M., Attias N., Avgar T. (2018). Moving in the Anthropocene: Global reductions in terrestrial mammalian movements. Science.

[88] Bailey D.W., Trotter M.G., Knight C.W., Thomas M.G. (2018). Use of GPS tracking collars and accelerometers for rangeland livestock production research. Transl. Anim. Sci..

[89] Manly B.F.J., McDonald L.L., McDonald T.L., Erickson W.P. (2002). Resource Selection by Animals.

[90] Johnson C.J., Nielson S.E., Merrill E.H., McDonald T.L., Boyce M.S. (2006). Resource selection functions based on use-availability data: Theoretical motivation and evaluation methods. J. Wildl. Manag..

[91] Skarin A., Danell O., Bergstrom R., Moen J. (2008). Summer habitat preferences of GPS-collared reindeer Rangifer tarandus tarandus. Wildlife Biol..

[92] Falu E.M.D., Brizuela M.A., Cid M.S., Cibils A.F., Cendoya M.G., Bendersky D. (2014). Daily feeding site selection of cattle and sheep co-grazing a heterogeneous subtropical grassland. Livest. Sci..

[93] Panzacchi M., Van Moorter B., Strand O., Loe L.E., Reimers E. (2015). Searching for the fundamental niche using individual-based habitat selection modelling across populations. Ecography.

[94] Spedener M., Tofastrud M., Devineau O., Zimmermann B. (2019). Microhabitat selection of free-ranging beef cattle in south-boreal forest. Appl. Anim. Behav. Sci..

[95] Tofastrud M., Devineau O., Zimmermann B. (2019). Habitat selection of free-ranging cattle in productive coniferous forests of south-eastern Norway. For. Ecol. Manag..

[96] Ager A.A., Johnson B.K., Kern J.W., Kie J.G. (2003). Daily and seasonal movements and habitat use by female rocky mountain elk and mule deer. J. Mammal..

[97] Taylor D.B., Schneider D.A., Brown W.Y., Price I.R., Trotter M.G., Lamb D.W., Hinch G.N. (2011). GPS observation of shelter utilisation by Merino ewes. Anim. Prod. Sci..

[98] Dolev A., Henkin Z., Brosh A., Yehuda Y., Ungar E.D., Shabtay A., Aharoni Y. (2014). Foraging behaviour of two cattle breeds, a whole-year study: II. Spatial distribution by breed and season. J. Anim. Sci..

[99] Liao C., Clark P.E., Shibia M., DeGloria S.D. (2018). Spatiotemporal dynamics of cattle behaviour and resource selection patterns on East African rangelands: Evidence from GPS-tracking. Int. J. Geogr. Inf. Sci..

[100] Benhamou S., Riotte-Lambert L. (2012). Beyond the Utilization Distribution: Identifying home range areas that are intensively exploited or repeatedly visited. Ecol. Modell..

[101] Skarin A., Sandström P., Alam M. (2018). Out of sight of wind turbines—Reindeer response to wind farms in operation. Ecol. Evol..

[102] Calcante A., Tangorra F.M., Marchesi G., Lazzari M. (2014). A GPS/GSM based birth alarm system for grazing cows. Comput. Electron. Agric..

[103] DeMars C.A., Auger-Méthé M., Schlägel U.E., Boutin S. (2013). Inferring parturition and neonate survival from movement patterns of female ungulates: A case study using woodland caribou. Ecol. Evol..

[104] Langrock R., Hopcraft J.G.C., Blackwell P.G., Goodall V., King R., Niu M., Patterson T.A., Pedersen M.W., Skarin A., Schick R.S. (2014). Modelling group dynamic animal movement. Methods Ecol. Evol..

[105] Harris N.R., Johnson D.E., McDougald N.K., George M.R. (2007). Social associations and dominance of individuals in small herds of cattle. Rangel. Ecol. Manag..

[106] Aharoni Y., Dolev A., Henkin Z., Yehuda Y., Ezra A., Ungar E.D., Shabtay A., Brosh A. (2013). Foraging behaviour of two cattle breeds, a whole-year study: I. Heat production, activity, and energy costs. J. Anim. Sci..

[107] Beker A., Gipson T.A., Puchala R., Askar A.R., Tesfai K., Detweiler G.D., Asmare A., Goetsch A.L. (2010). Energy Expenditure and Activity of Different Types of Small Ruminants Grazing Varying Pastures in the Summer. J. Appl. Anim. Res..

[108] Cavalcanti S.M.C., Gese E.M. (2010). Kill rates and predation patterns of jaguars (*Panthera onca*) in the southern Pantanal, Brazil. J. Mammal..

[109] Laporte I., Muhly T.B., Pitt J.A., Alexander M., Musiani M. (2010). Effects of Wolves on Elk and Cattle Behaviours: Implications for Livestock Production and Wolf Conservation. PLoS ONE.

[110] Steyaert S.M.J.G., Støen O.-G., Elfström M., Karlsson J., Lammeren R.V., Bokdam J., Zedrosser A., Brunberg S., Swenson J.E. (2011). Resource selection by sympatric free-ranging dairy cattle and brown bears Ursus arctos. Wildlife Biol..

[111] Sivertsen T.R. (2017). Risk of Brown Bear Predation on Semi-Domesticated Reindeer Calves–Predation Patterns, Brown Bear–Reindeer Interactions and Landscape Heterogeneity. Ph.D. Thesis.

[112] Karlsson J., Støen O.-G., Segerström P., Stokke R., Persson L.-T., Stokke  L.-H., Persson S., Stokke N., Persson A., Segerström E. (2012). Björnpredation På Ren och Potentiella Effekter Av Tre Förebyggande Åtgärder.

[113] Betteridge K., Costall D., Balladur S., Upsdell M., Umemura K. (2010). Urine distribution and grazing behaviour of female sheep and cattle grazing a steep New Zealand hill pasture. Anim. Prod. Sci..

[114] Virgilio A.d., Morales J.M., Lambertucci S.A., Shepard E.L.C., Wilson R.P. (2018). Multi-dimensional Precision Livestock Farming: A potential toolbox for sustainable rangeland management. PeerJ.

[115] D’Eon R.G., Serrouya R., Smith G., Kochanny C.O. (2002). GPS Radiotelemetry Error and Bias in Mountainous Terrain. Wildl. Soc. Bull..

[116] Frair J.L., Nielsen S.E., Merrill E.H., Lele S.R., Boyce M.S., Munro R.H.M., Stenhouse G.B., Beyer H.L. (2004). Removing GPS collar bias in habitat selection studies. J. Appl. Ecol..

[117] Frair J.L., Fieberg J., Hebblewhite M., Cagnacci F., DeCesare N.J., Pedrotti L. (2010). Resolving issues of imprecise and habitat-biased locations in ecological analyses using GPS telemetry data. Philos. Trans. R. Soc. Lond. B Biol. Sci..

[118] Dussault C., Courtois R., Ouellet J.-P., Huot J. (2001). Influence of Satellite Geometry and Differential Correction on GPS Location Accuracy. Wildlife Soc. Bull..

[119] Swain D.L., Wark T., Bishop-Hurley G.J. (2008). Using high fix rate GPS data to determine the relationships between fix rate, prediction errors and patch selection. Ecol. Modell..

[120] Wilson R.P., Liebsch N., Davies I.M., Quintana F., Weimerskirch H., Storch S., Lucke K., Siebert U., Zankl S., Muller G. (2007). All at sea with animal tracks; methodological and analytical solutions for the resolution of movement. Deep Sea Res. Part II Top. Stud. Oceanogr..

[121] McDonnell S., Torcivia C. (2020). Preliminary Proof of the Concept of Wild (Feral) Horses Following Light Aircraft into a Trap. Animals.

[122] Nicas J. (2015). They’re Using Drones to Herd Sheep. The Wall street Journal. https://www.wsj.com/articles/theyre-using-drones-to-herd-sheep-1428441684.

[123] Brady H. (2017). Watch a Drone ‘Herd’ Cattle across Open Fields. National Geographic. https://www.nationalgeographic.com/news/2017/08/drone-herd-cattle-field-california-video-spd/.

[124] Brunberg E., Eythórsdóttir E., Dýrmundsson Ó.R., Grøva L. (2020). The presence of Icelandic leadersheep affects flock behaviour when exposed to a predator test. Appl. Anim. Behav. Sci..

[125] Umstätter C. (2011). The evolution of virtual fences: A review. Comput Electron. Agric..

[126] Anderson D.M., Estell R.E., Holechek J.L., Ivey S., Smith G.B. (2014). Virtual herding for flexible livestock management–a review. Rangel. J..

[127] Umstätter C., Morgan-Davies J., Waterhouse T. (2015). Cattle responses to a type of virtual fence. Rangel. Ecol. Manag..

[128] Bishop-Hurley G.J., Swain D.L., Anderson D.M., Sikka P., Crossman C., Corke P. (2007). Virtual fencing applications: Implementing and testing an automated cattle control system. Comput. Electron. Agric..

[129] Markus S.B., Bailey D.W., Jensen D. (2014). Comparison of electric fence and a simulated fenceless control system on cattle movements. Livest. Sci..

[130] Umstätter C., Brocklehurst S., Ross D.W., Haskell M.J. (2013). Can the location of cattle be managed using broadcast audio cues?. Appl. Anim. Behav. Sci..

[131] Lee C., Henshall J.M., Wark T.J., Crossman C.C., Reed M.T., Brewer H.G., O’Grady J., Fisher A.D. (2009). Associative learning by cattle to enable effective and ethical virtual fences. Appl. Anim. Behav. Sci..

[132] Campbell D.L.M., Lea J.M., Farrer W.J., Haynes S.J., Lee C. (2017). Tech-Savvy Beef Cattle? How heifers respond to moving virtual fence lines. Animals.

[133] Campbell D.L.M., Lea J.M., Haynes S.J., Lea J.M., Farrer W.J., Leigh-Lancaster C.J., Lee C. (2018). Virtual fencing of cattle using an automated collar in a feed attractant trial. Appl. Anim. Behav. Sci..

[134] Campbell D.L.M., Haynes S.J., Lea J.M., Farrer W.J., Lee C. (2019). Temporary exclusion of cattle from a riparian zone using virtual fencing technology. Animals.

[135] Jouven M., Leroy H., Ickowicz A., Lapeyronie P. (2012). Can virtual fences be used to control grazing sheep?. Rangel. J..

[136] Marini D., Llewellyn R., Belson S., Lee C. (2018). Controlling within-field sheep movement using virtual fencing. Animals.

[137] Marini D., Meuleman M.D., Belson S., Rodenburg T.B., Llewellyn R., Lee C. (2018). Developing an ethically acceptable virtual fencing system for sheep. Animals.

[138] Brunberg E., Bøe K.E., Sørheim K.M. (2015). Testing a new virtual fencing system on sheep. Acta. Agric. Scand. A Anim. Sci..

[139] Brunberg E.I., Bergslid I.K., Bøe K.E., Sørheim K.M. (2017). The ability of ewes with lambs to learn a virtual fencing system. Animal.

[140] Fay P.K., McElligott V.T., Havstad K.M. (1989). Containment of free-ranging goats using pulsed-radio-wave- activated shock collars. Appl. Anim. Behav. Sci..

[141] Eftang S., Bøe K.E. (2017). Bruk av Nofence Virtuelt Gjerde til Geit i et Dyrevelferdsperspektiv.

[142] Lee C., Colditz I.G., Campbell D.L.M. (2018). A framework to assess the impact of new animal management technologies on welfare: A case study of virtual fencing. Front. Vet. Sci..

[143] Ledgerwood D.N., Winckler C., Tucker C.B. (2010). Evaluation of data loggers, sampling intervals, and editing techniques for measuring the lying behaviour of dairy cattle. J. Dairy Sci..

[144] Zobel G., Weary D.M., Leslie K., Chapinal N., von Keyserlingk M.A.G. (2015). Technical note: Validation of data loggers for recording lying behaviour in dairy goats. J. Dairy Sci..

[145] Lind A.-K., Lindahl C. (2018). Moocall-En Sensor Med Koll på Kalvningar.

[146] Wiklund E., Malmfors G., Lundström K., Rehbinder C. (1996). Pre-slaughter handling of reindeer bulls (Rangifer tarandus tarandus L.)—Effects on technological and sensory meat quality, blood metabolites and muscular and abomasal lesions. Rangifer.

[147] Kearton T., Marini D., Cowley F., Belson S., Lee C. (2019). The effect of virtual fencing stimuli on stress responses and behaviour in sheep. Animals.

[148] McDonald C.L., Beilharz R.G., McCutchan J.C. (1981). Training cattle to control by electric fences. Appl. Anim. Ethol..

